# One Health-ness Evaluation of Cysticercosis Surveillance Design in Portugal

**DOI:** 10.3389/fpubh.2018.00074

**Published:** 2018-03-13

**Authors:** Ana Gloria Fonseca, Jorge Torgal, Daniele de Meneghi, Sarah Gabriël, Ana Cláudia Coelho, Manuela Vilhena

**Affiliations:** ^1^Public Health Department, NOVA Medical School, NOVA University of Lisbon, Lisbon, Portugal; ^2^Department of Veterinary Science, University of Turin, Grugliasco, Turin, Italy; ^3^Department of Veterinary Public Health and Food Safety, Faculty of Veterinary Medicine, Ghent University, Ghent, Belgium; ^4^Escola de Ciências Agrárias e Veterinárias, Universidade de Trás os Montes e Alto Douro (UTAD), Vila Real, Portugal; ^5^Instituto de Ciências Agrárias e Ambientais Mediterrânicas (ICAAM), Universidade de Évora, Évora, Portugal

**Keywords:** One Health, case study, cysticercosis, evaluation, surveillance, neglected disease

## Abstract

The increasing occurrence of human cysticercosis, a zoonotic neglected disease, is challenging the traditional prevention and control paradigm and calling for One Health (OH) solutions in industrialized countries. OH solutions for health interventions are increasingly being used to capture expected and unexpected outcomes across people, animals, and the environment. The Network for Evaluation of One Health (NEOH) proposes an evidence-based framework, relying on systems and mixed methods approaches to evaluate the One Health-ness. In this case study, this tool is used to evaluate the design of the Observatory of Taeniasis and Cysticercosis, as an example of intersectorial collaboration for surveillance in Portugal. The OH Initiative (drivers and expected outcomes) and its system (boundaries, aim, dimensions, actors, and stakeholders) were described. The different aspects of this Initiative were scored with values from 0 (=no OH approach) to 1 (=perfect OH approach). The OH index was 0.31. Its OH ratio is 1.98. Overall scores were as follows: OH thinking 0.75; OH planning 0.60; OH working 0.60; OH sharing 0.35; OH learning 0.50; and systemic organization 0.50. Operational levels of the Initiative are the main strengths, indicating a comprehensive multidimensional innovative approach and transdisciplinarity. Critical issues in the supporting infrastructure were observed, related to communication, learning and organizational gaps in the project, with the evaluation being conducted as the project is being designed and implemented. The strengths and weaknesses detected may be used to refine the Initiative. This case study therefore exemplifies and supports OH assessment also for ongoing projects, at design and early implementation stages for guiding and guaranteeing an OH-oriented perspective.

## Introduction

In European countries, emerging *Taenia solium* taeniasis and cysticercosis in urban areas is questioning the traditional disease paradigm as a zoonotic disease of developing countries, requesting for integrated approaches and One Health (OH) solutions (1, 2). Human cysticercosis is a preventable fecal–oral transmitted neglected parasitic infection caused by cysticerci of the tapeworm *T. solium*. Neurocysticercosis (NCC), the single major cause of adult onset seizures in endemic areas, occurs when cysticerci lodge in the central nervous system in humans (3).

In non-endemic settings, imported and autochthonous cysticercosis emergence is plausible, impacting on human, animal, and environmental health and welfare. In Portugal, 357 hospitalized NCC cases were detected during a retrospective study covering the 2006–2013 period (4). In the absence of legal notification or established surveillance system, the Observatory of Taeniasis and Cysticercosis (OTC) was designed.

The OTC aims for an OH-based national disease surveillance system, increasing knowledge and awareness for evidence-based interventions leading to disease control and eradication, based on sustainable solutions impacting on human, animal, environmental, societal, and economic health and welfare. The surveillance design is based on cross-sectorial and transdisciplinary collaboration between human and animal health institutions at local, regional, and national levels, targeting humans and pigs, aiming at detecting, and treating the tapeworm carriers, therefore reducing the risk of infection in the human and pig populations.

The OTC was evaluated using the innovative framework developed by the Network for Evaluation of One Health (NEOH), representing a case study illustrating the application of this methodology and grasping to what extent the underlying integration as a principle and approach is actually contributing to manage the health problem (5, 6).

## Background and Rationale

In the *T. solium* life cycle and transmission, both pig and humans can be infected by the metacestode larval stages and develop disease. The infection results from the ingestion of *T. solium* eggs, shed in the feces of human tapeworm carriers, through contaminated food and water or by autoinfection: the eggs hatch, migrate, and lodge in the tissues, where they develop into cysticerci. Human become tapeworm carriers by ingesting undercooked pork meat containing viable cysticerci in muscle tissue. Cysticercosis clinical symptoms often appear months/years after the infection. The acute and chronic neurological symptoms/disease in humans determines health-care costs and suffering in the form of disease-adjusted life years.

Human cysticercosis has been primarily associated with rural and peri-urban areas of developing countries where sanitation is poor and pigs roam freely and eat human feces. In industrialized countries, cysticercosis, although rare, is increasingly being diagnosed and has been associated with migration and travel from endemic areas. However, it may occur in individuals with no history of pork consumption or travel to endemic areas (3, 7). Epidemiological studies have demonstrated tight clustering in households and suggest the most common source of infective eggs is an asymptomatic household tapeworm carrier (8).

In Portugal, cysticercosis was considered prevalent before 1975. Thereafter, improved sanitation and industrialized pig production under veterinary control made Portugal officially free of porcine cysticercosis; diagnosed human cases have been considered mainly imported (Statistics Portugal, www.ine.pt). As in most European countries, there is no NCC notification or surveillance. There is scarce and not updated data on human and porcine cysticercosis. While old, mostly rural human cases indicated probable autochthonous infection; nowadays, industrialized pig production in closed systems under veterinary control is rather generalized. Human disease cases tend to occur in 20–64 years age group (55.2%) in urban settings, indicating imported cases or the presence of tapeworm carriers infecting other people, without the presence of infected pig intermediate hosts (4).

Thus far, the disease control model has reflected a rural reality, where free-ranging pigs are raised, sanitation conditions are poor, and sanitary inspection measures are insufficient. Control tools generally include pig-oriented measures, human tapeworm treatments, health education, and sanitary improvements. Environmental contamination with *Taenia* spp. eggs is a key issue in most studies, influencing the presence of *Taenia* spp. antigens in both pigs and humans (9). Soil-related, socioeconomic, and behavioral factors are associated with the emergence of significant clustering of human cysticercosis (8, 9). However, very few studies have been produced in urban environment of developed countries. In this setting, transmission patterns are likely to relate more with behavior, housing conditions, water supply, basic sanitation, schooling and birthplace of the individual or relatives, human migration patterns, and food preparation; the role of free roaming pigs or soil conditions not being as obvious (10).

### OTC in Portugal: The OH Initiative

The OTC was designed, aiming at the surveillance of taeniasis and cysticercosis, fostered by human health and animal health national authorities in collaboration, asking for cross-sectorial, inter, and transdisciplinary cooperation and networking at all stages of development and action. It aims to obtain essential information on the burden and epidemiology of cysticercosis in Portugal. Its final aim is sustainable health protection through cysticercosis prevention and control, focusing on human health and welfare without disregarding animal health and the ecosystem.

Main drivers of the Initiative are as follows: (i) the disease occurrence in migrants from endemic areas, whose access to health care may be compromised by legal and economic issues (illegal immigration and poverty) and, rarely, in non-migrants: 45 hospitalizations per year (4, 11–14); (ii) the unawareness of the disease burden due to absence of (systematic) human cysticercosis and pig cysticercosis data; (iii) the non-applicability of the traditional disease prevention and control tools (involving the pig as an intermediate host) to the current disease scenario (human-to-human transmission); and (iv) the fact that the disease can be effectively prevented by tapeworm eradication in human carriers (that is, preventing transmission).

The Initiative comprises the following core surveillance activities:

(1) Baseline characterization and thereafter monitoring of the national epidemiological scenario, by systematically obtaining and analyzing the available disciplinary administrative data, to identify possible geographic hotspots: (a) human health sector: human cysticercosis hospitalizations (Hospital Episodes Statistics); (b) animal health sector: pig cysticercosis diagnosis at slaughterhouses and at non-industrial pig distribution units (less than 100 animals); and (c) human social sector: Portuguese resident and foreign resident population data (Statistics of Portugal).
(2) In the geographic hotspots, implementing a questionnaire-based surveillance targeting health-care units to detect and monitor new human cysticercosis diagnosis (notification system) associated with an epidemiologic field survey to patients and contacts, exploring human, animal, and environmental factors, identifying possible human tapeworm carriers. It uses the structural resources and communication algorithms already in practice for other reportable diseases (national notifiable disease surveillance system) under the aegis of the public health national authority. It involves hospital and primary care physicians as primary reporting source and public health physicians at the local health department as primary level data recipients and reporting source for the epidemiologic field survey. Secondary level data recipients are national health authority and OTC.
(3) In the geographic hotspots, implementing a questionnaire-based surveillance targeting non-industrial porcine distribution units (less than 100 animals) to detect and monitor new pig cysticercosis diagnosis at slaughterhouses (notification system) associated with an epidemiologic field survey to the pig handlers, exploring human, animal, and environmental factors, identifying possible human tapeworm carriers. It uses the structural resources and communication algorithms already in practice for other reportable diseases under the aegis of animal health and food national authority and establishing a structured communication pathway between animal health and human health sectors in the reporting and surveillance system. It involves slaughterhouse inspectors as primary reporting source and public human health physicians at the local health department as primary level data recipients and reporting source for the epidemiologic field survey. Secondary level data recipients are the national health authority and the OTC.
(4) Biological species diagnosis of tapeworm carriers in the reference laboratory, using *T. solium* taeniasis coproantigen detection test with molecular species confirmation among human cysticercosis cases and human case contacts identified in (2) and (3). Referral for laboratorial diagnosis is done through the local health department, involving primary care/hospital physicians and public health physicians, the reference laboratory also acting as a reporting source in the surveillance system. Secondary level data recipients are the national health authority and the OTC.
(5) Hospital-based treatment for the identified human tapeworm carriers within the local health-care system. Referral for treatment is done upon laboratorial diagnosis through the local health department, involving primary care/hospital physicians and public health physicians.
(6) Monitoring, stewardship and supervision of the reporting process, the field epidemiological surveys, and the referral for laboratorial diagnosis and treatment.
(7) Yearly data management and analysis and report generation and dissemination, complying with the national standards of confidentiality, to the involved actors, stakeholders, and decision-makers.

The Initiative is led by human and animal health sectors in coordination under the aegis of Human and Animal Health Authorities. It fosters trans-sectorial communication and data sharing at different hierarchical and decision levels: human and animal health. Within each sector, the hierarchical channels and structural elements already in use for other purposes, namely, disease notification, are used. Innovation comes from (i) introducing human and pig cysticercosis as a notifiable disease and within the same surveillance process; (ii) triggering active human case detection (human tapeworm carrier) upon the diagnosis of a case of human or pig cysticercosis, active animal case detection in the affected non-industrial pig distribution unit also being also promoted; (iii) promoting detection and correct medical approach of tapeworm carriers, paving the way for control and eradication of human-to-human transmission (NCC disease transmission control); (iv) enforcing laboratorial technology, introducing the use of specific diagnostic tests beyond research purposes; (v) enforcing ongoing transdisciplinary and cross-sectorial networking, feedback, and collaboration; and (vi) harboring academic and field research purposes, through collaboration with postgraduate educational institutions.

The expected consequences of this Initiative are improvements at the level of the disease burden estimates, as well as at the level of the epidemiological and medical approach of each individual case, impacting on human, animal, and environmental health.

Planning surveillance should be an iterative process, requiring the regular reassessment of objectives and methods, to double check if the purposes of the surveillance system are being met. To gather consistent and systematic awareness data and identify the changes related to the organization of services, information systems, and institutional relationships, allowing for a complete outcome-based evaluation, the Initiative needs to be running 5 years minimum.

The Initiative was, however, designed considering OH concerns and approach to surveillance and problem solving. Therefore, OTC design was evaluated for its OH characteristics and One Health-ness (OH-ness).

The adhesion to OH protocol is often praised but not proved. The evaluation of OH-ness is hence an innovation in the OH context and a preliminary step to assess the real advantages of OH in comparison with the traditional approaches to health evaluation.

The evaluation question was the following: is the OTC designed according to OH characteristics and requirements? This evaluation is being conducted as the OTC is being implemented, in a feedback loop that allows for ongoing design and implementation readjustments to ensure effective and efficient OH action, in theory and practice. End users are the Human and Animal Health Directorates and the OTC itself. The Initiative and OH evaluation may provide an example of cross-sectorial and transdisciplinary collaboration at national level on how to manage a non-notifiable neglected zoonosis in a European country.

## Methods

For taeniasis and cysticercosis surveillance design evaluation, the Network for Evaluation of One Health (NEOH) evaluation framework was used (5). It uses a systems approach and aims to relate the OH process characteristics (OH-ness), namely, operational aspects (thinking, planning, and working) and supporting infrastructure aspects (sharing, learning, and systemic organization). It consists of a mix methods approach, including a descriptive and qualitative assessment with a semiquantitative scoring for the evaluation of the degree and structural balance of OH-ness. The six different aspects for a perfect OH approach of the Initiative were scored using standardized aspect-specific assessment tools: OH thinking, OH planning, OH working, OH sharing, OH learning, and systemic organization. Each one consisted of a series of up 17 questions and an associated scoring system with values between 0 and 1 as well as spider diagrams, with a score of 1 reflecting a full realization of the different OH characteristics (ideal scenario). OH thinking assesses the way actors and stakeholders think in and about the OH Initiative and the system in which it operates (the context) [scoring criteria: dimension coverage and balance; Initiative to environment match; integrative health approach; system features and target; sustainability and social–ecological considerations; perspectives and theory of change (TOC) factors]. OH planning evaluates planning and resource allocations in the Initiative (scoring criteria: common aims; stakeholder and actor engagement; self-assessment and plan revisions; and individual objectives). OH working assesses the interdisciplinary and participatory engagement in OH Initiatives (scoring criteria: broadness; collaboration; transdisciplinary balance; cultural and social balance; and flexibility and adaptation). OH sharing evaluates the extent and methods of information and data sharing infrastructures in OH Initiatives (scoring criteria: general information and awareness sharing; data and information sharing; methods and results sharing; and institutional memory and resilience to change). OH learning evaluates the learning infrastructure of the Initiative (scoring criteria: focus on adaptive and generative individual learning; focus on adaptive and generative team learning; adaptive and generative organizational learning; direct learning environment supportive of adaptive and generative learning; and general learning environment supportive of adaptive and generative learning). System organization assesses the systemic organization of the Initiative, focusing on leadership skills and criteria for effective teamwork (scoring criteria: team structure; social and leadership structures and skills; competence; and focus on innovation). Detailed scoring criteria, metrics, and results can be found in Supplementary Material.

The scores for each assessed OH aspect were plotted on to the spokes of a spider diagram to allow visualization of the overall project integration and balance between operational and infrastructure aspects. These scores were combined into quantitative OH index (OHI) and OH ratio (OHR) for a holistic appreciation. The OHI, reflecting the degree of integration of the operational aspects and infrastructure, was calculated from the area enclosed by the points when plotted onto de spider diagram according to the following equation:

$$\text{OHI}=\frac{\left\{\begin{array}{c} \left(\text{ScP}\times \text{ ScT})\;+\;(\text{ScL}\times \text{ScP})\;+\;(\text{ScS}\times \text{ScL})+\;(\text{ScO}\times \text{ScS}\right)\\ +\;(\text{ScW}\times \text{ScO})\;+\;(\text{ScT}\times \text{ScW}) \end{array}\right\}}{6}$$

where ScP is the score obtained in OH planning, ScT is the score obtained in OH thinking, ScL is the score obtained in OH learning, ScS is the score obtained in OH Sharing, ScO is the score from systemic organization, and ScW is the score obtained in OH working.

The OHR, reflecting the balance between operations and infrastructure, was calculated dividing the area enclosed by the points associated with OH operations by that associated with OH infrastructure, according to the following equation:

$$\text{OHR}=\frac{\left(\frac{\text{ScO}\times {\text{ScW}}^{2}}{\text{ScO}+\text{ScW}}\right)+(\text{ScW}+\text{ScT})+(\text{ScT}+\text{ScP})+\left(\frac{{\text{ScP}}^{2}\times \text{ScL}}{\text{ScP}+\text{ScL}}\right)}{\left(\frac{\text{ScP}\times {\text{ScL}}^{2}}{\text{ScP}+\text{ScL}}\right)+(\text{ScL}+\text{ScS})+(\text{ScS}+\text{ScO})+\left(\frac{{\text{ScO}}^{2}\times \text{ScW}}{\text{ScO}+\text{ScW}}\right)}.$$

The OH-ness evaluation was preceded by the system identification considering the OTC as a subsystem and the outline of the expected outcome based on the TOC of the Initiative, as proposed by the NEOH evaluation framework. The proposed quali-quantitative evaluation of OH-ness, also in combination with other evaluation approaches (e.g., a process evaluation or a cost–benefit evaluation) enables the detection of strengths and weaknesses of the Initiative. The combination of the six OH characteristics in single quantitative OHI and OHR associated with visual images contributes to objectivity when considering two or more OH Initiatives, although an optimal range for OHI, and not a higher value, may be indicative of a “better” OH Initiative (5).

The scope of the evaluation and delimitation of the system are considered pivotal for the outcome of the evaluation. The rationale behind this case study and the application of the evaluation framework were not to ascertain whether an already fully established and implemented Initiative meets OH characteristics, but rather to ensure that a newly started Initiative will achieve this on the longer term, by correcting its design on the way. The evaluation type was process self-evaluation. Three internal evaluators conducted the evaluation, and three external evaluators completed a general review of the evaluation. Data and information gathering relied mainly on focus group discussions and project documents.

## Results

### Identification of the System and TOC

The system includes the OTC (OH Initiative) as a subsystem operating within the system, aiming at surveillance of taeniasis and cysticercosis, impacting on human, animal, and also environmental health.

System boundaries are determined by the following considerations:

(i) Cysticercosis is emerging in European non-endemic countries, affecting humans, pigs, and environmental surroundings. Unawareness of disease risk, communicability, and costs is recognized. The Initiative is being set at national level, but the results may be valuable at European and International levels. These elements allow the identification of main geographical, life, and knowledge dimensions of the system.
(ii) The disease (NCC) may be asymptomatic for long periods of time and may be associated with long-term sequela and disability. It can be acquired in foreign endemic countries, and human tapeworm carriers are usually asymptomatic, thus favoring transmission. Any changes within the system will require years, all this allowing the identification of the main time dimension of the system and adding complexity to knowledge, life, and management dimensions.
(iii) The disease is preventable. However, there is no established surveillance for human disease, although it exists for other communicable diseases, and surveillance performs deficiently for animal disease. Hence, the importance legislative dimension of the system. The existing surveillance framework is disciplinary and sectorial in nature, cysticercosis successful surveillance and control requiring networking between actors and stakeholders at different organizational and operational levels. It brings together public health and veterinary authorities, academia, laboratories and hospitals, and medical and veterinary practitioners. These elements allow the identification of legislative and networking dimensions, also reinforcing management dimensions. A possible long-term impact on economy needs to be taken into account (economic dimension).

The actors and stakeholders are Human Health Directorate, Animal and Food Directorate, human and veterinary health academic institutions, public health and health care-providing institutions (hospitals and primary care facilities), practitioners in human and animal health, and laboratories in human and animal health. Stakeholders also include the community, patients, and political decision-makers.

**Figure 1:**
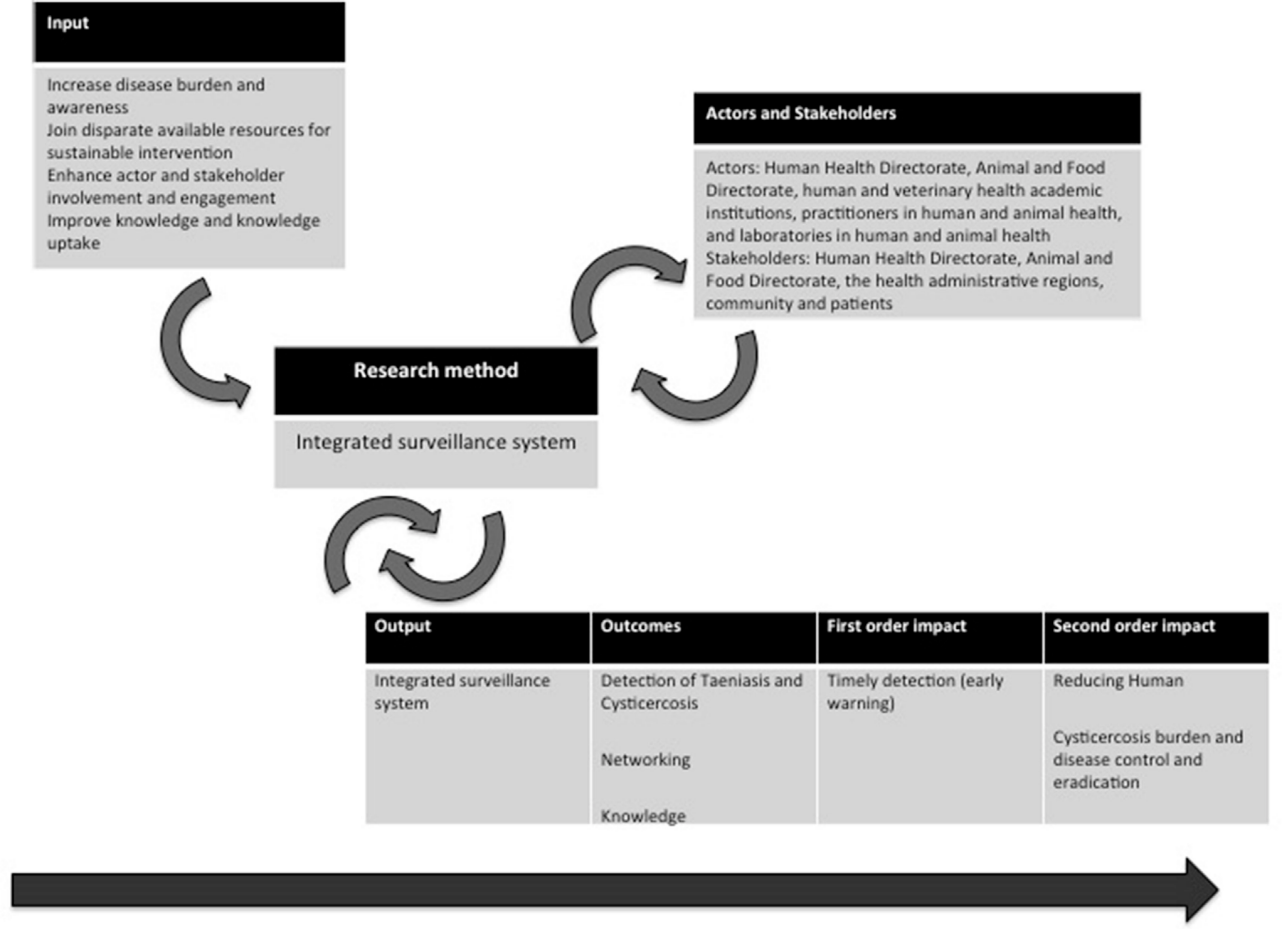
Theory of change applied to the Observatory of Taeniasis and Cysticercosis in Portugal.

The TOC for this case study is the awareness and knowledge on the epidemiology of cysticercosis in Portugal (Figure 1). In European countries, namely, Portugal, where basic sanitation and food chain quality control have long been achieved and consolidated, disease can be controlled and eradicated by efficient and correct identification and treatment of human tapeworm carriers. This can be achieved through an integrated human and animal health surveillance system that combines passive and active surveillance activities. Ecological and environmental variables favoring transmission are considered within these activities and subsequent intervention. The enhanced networking, knowledge, and results will allow greater collaboration with health systems at international level and the adoption of preventive measures, guiding future more informed and productive research and control solutions in a global space. Moreover, it will be of interest for national and European governments and policy makers.

Cysticercosis surveillance being an ongoing process at early implementation, only expected outcomes are outlined. Disciplinary outcomes include the following: (i) disease awareness and determinants definition; (ii) disease burden (frequency, severity, health status, etc.); (iii) tapeworm carriers detected; and (iv) effective tapeworm carriers’ treatments. Interdisciplinary outcomes are based on the Initiative innovation allowing for reporting practices, generation of new sources of data and networks, also creating a favorable environment for developing new operational projects, research projects, and national and international networking and collaboration. Also, the development of guidelines concerning case diagnosis, epidemiological case contacts’ survey, and treatment is an interdisciplinary outcome. The evolving trans-sectorial and transdisciplinary official and non-official collaboration and transparency at different levels and hierarchies within the system as well as the expected increased interest in OH approaches for solving complex health problems are OH outcomes of the Initiative. Moreover, innovation is brought by introducing a new model for control of a neglected disease in European settings, involving changing the disease control paradigm.

### OH-ness

**Figure 2:**
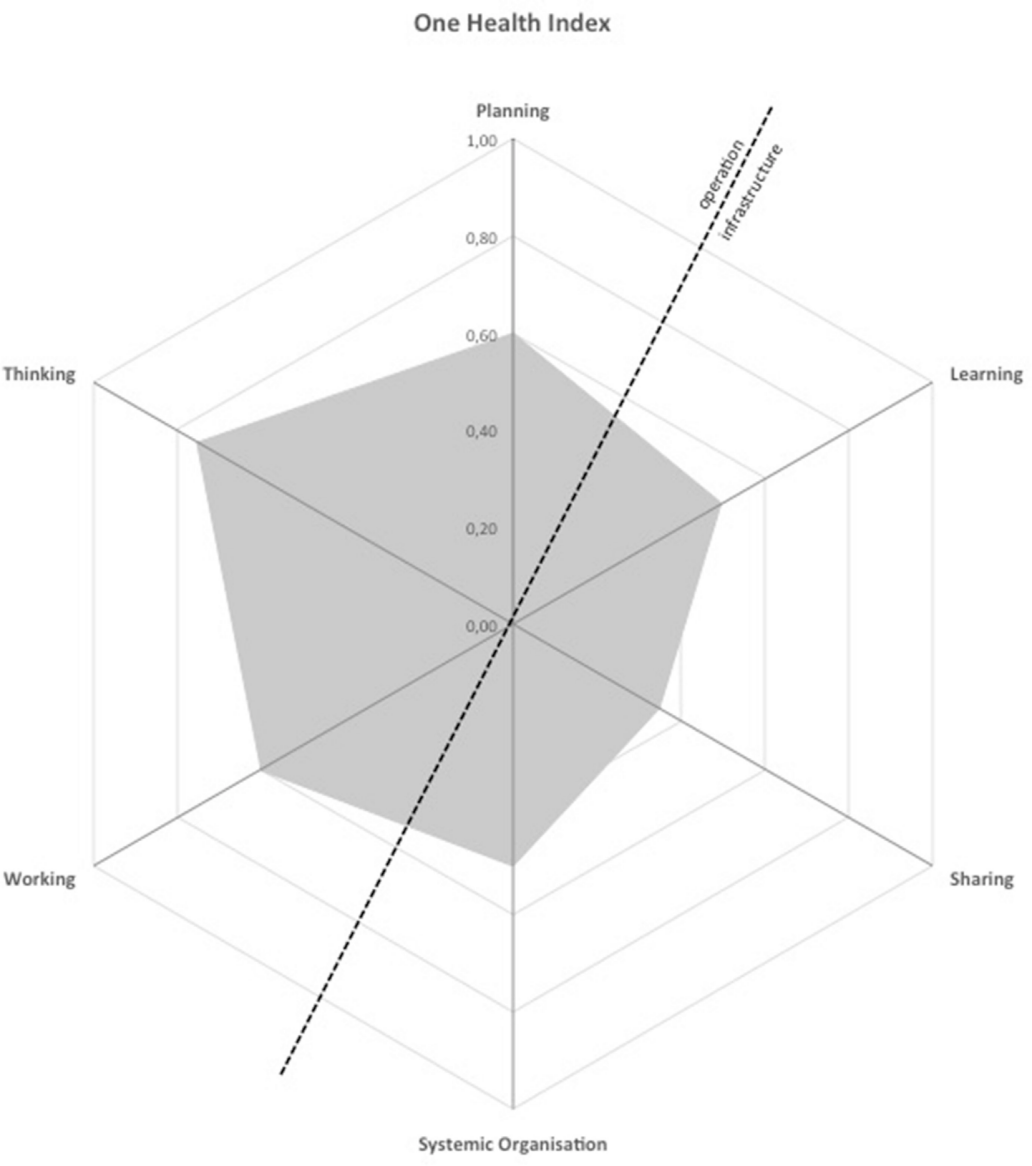
Spider diagram illustrating the degree of One Health implementation and the balance between the operational and the supporting means of the Observatory of Taeniasis and Cysticercosis in Portugal.

The OH index was 0.31. Its OHR is 1.98. The results were depicted in a spider diagram with the surface area and shape illustrating the degree of OH implementation and the balance between the operational and the supporting means (Figure 2). The operational levels of the Initiative are the main strengths, namely, OH thinking and OH working, indicating a comprehensive multidimensional approach and transdisciplinarity. On the other hand, weaknesses in the supporting infrastructure, namely, OH sharing, team structure, and organizational learning were observed.

One Health thinking score was 0.75. Eight different dimensions were considered: space, time, life, knowledge, and network were considered crucial dimensions and obtained the highest score (=1), followed by economy (=0.4), and the passive recognition of management and legislative dimensions (=0.2). The scorings of the considered criteria were as follows: dimension coverage and balance 0.8; Initiative to environment match 0.7; integrative health approach 1.0; system features and target 0.7; sustainability and social–ecological considerations 0.8; and perspectives and TOC factors 0.4.

One Health planning score was 0.60. It was calculated according to the match between tasks, resources, and responsibilities. The scorings of the considered criteria were as follows: common aims 1.0; stakeholder and actor engagement 0.7; self-assessment and plan revisions 0.6; and individual objectives 0.4–1.

One Health working score was 0.60. The scorings of the considered criteria were as follows: broadness of the Initiative 0.8; collaboration 0.6; transdisciplinary balance 0.8; cultural and social balance 0.5; and flexibility and adaptation 0.8.

One Health sharing score was 0.35, reflecting overall data and information sharing infrastructure. The scorings of the considered criteria were as follows: general information and awareness sharing 0.3; data and information sharing 0.4; methods and results sharing 0.8; and institutional memory and resilience to change 0.1.

One Health learning score was 0.50, and it assesses the learning infrastructure. The scorings of the considered criteria were as follows: focus on adaptive and generative individual learning 0.5; focus on adaptive and generative team learning 0.7; adaptive and generative organizational learning 0.4; direct learning environment supportive of adaptive and generative learning 0.4; and general learning environment supportive of adaptive and generative learning 0.5.

Systemic organization score was 0.50, the tool focusing on leadership skills and criteria for effective teamwork. The scorings of the considered criteria were as follows: team structure 0.5; social and leadership structures and skills 0.7; competence 0.5; and focus on innovation 1.0.

## Discussion

The establishment of integrated surveillance programs focusing on tapeworm carriers’ detection and treatment, for disease control and eradication, as the OTC program, may effectively tackle cysticercosis in industrialized countries. Adhesion to OH standards should guide surveillance program development at all stages. OH evaluation tools may play a role in certifying and guaranteeing adhesion.

In the evaluation performed, operational aspects were the main strengths (OH thinking, OH working, and OH planning) indicating a comprehensive transdisciplinary-integrated health approach, sustainability, and ecological consideration. Weakness in the infrastructure and supporting means for the Initiative (OH learning, OH sharing, and systemic organization) was detected, the most critical being related to information and data sharing and team structures. The six assessments were combined in an OHI of 0.31, reflecting the degree of integration of operational and infrastructure aspects of the Initiative, given by the proportion of the surface of the spider diagram hexagon covered (Figure 2). OHR was 1.98, reflecting the balance between operational and infrastructure aspects of the Initiative through its symmetry over the diagonal on the spider diagram (Figure 2). The optimal range for OHI and OHR has, however, not been established (5).

Considering the individual aspects assessment, the higher score was obtained for OH thinking (0.75). The Initiative is at designing and early implementation stages, the conceptualization of the Initiative and the thinking in an about the Initiative and the system being comparatively better consolidated. It has a highly integrated approach, covering diverse dimensions at differing scales and incorporating many perspectives. It aims to understand disease patterns and trends, identifying where to intervene to control disease transmission. It addresses mainly human, animal, and environmental concerns also being considered. It impacts on the tree pillars of sustainability (society, environment, and economy) although this cannot yet be captured. The knowledge resulting from the Initiative may apply internationally to countries with similar societal and epidemiological characteristics.

One Health planning aspect (0.60) analysis showed that essential stakeholders and sectorial and disciplinary actors are identified and described, but their full engagement is ongoing. The Initiative is planned to aim at sustainable health outcomes. Participating institutions from human and animal health sectors (national, regional, and local health authorities; health professionals working in human care, animal care and veterinary inspection; and academia and reference laboratory) and the formal and informal communication and networking generated are planned to achieve the aim. Planned formal and informal feedback communication loops and yearly formal reports’ discussion with the national health authorities and their disclosing to other stakeholders and actors within the Initiative allow for underway corrections of the planned activities and involvement of new actors and stakeholders as needed. For each of the planned activities, according to the formulated objectives, matching of roles, responsibilities, and competencies was clearly established but resource allocation is ongoing.

One Health working (0.60) assessed the transdisciplinarity and the degree of cross-disciplinary working and leadership enabling an innovative approach to the problem. A flexible coordination between professionals coming from animal health and human health areas was developed, the environmental component being developed within these areas. The Initiative is broad and cross-sectorial, although human health and animal health sectors dominate; disciplines, methods, and scales of analysis are diverse. Disciplines involved include clinical medicine, laboratory and diagnostics, public health, and epidemiology (including field and applied epidemiology) from academic and non-academic, governmental and non-governmental fields in the human and animal health sectors. There is a reasonable degree of interaction between actors from the different disciplines. There are difficulties in enforcing the methodology of the Initiative and in the communication between governmental institutions, the Initiative not being considered a priority. The aim seemed clear to all, there were face-to-face meetings, but part of its implementation still lacks the approval of government institutions. The methodology has been discussed with the actors and stakeholders, including the health authorities, and it has been adjusted to the work reality of each sector. Being a long-term systematic surveillance project at designing and early implementation phase, the project design can adapt to internal and external changes that may influence its implementation.

The lowest score obtained was in OH sharing (0.35). The Initiative does not yet have official mechanisms to facilitate sharing of information, although regular (annual) reports, newsletters, and workshops are to be implemented. Online and face-to-face meetings occur as deemed necessary. It uses peer-reviewed publications and conferences to share relevant data resulting in new knowledge production (4). Compliance with confidentiality and data protection issues need to be tackled since it may interfere with data sharing and accessibility. Within the Initiative, methods and results are to be discussed and shared. Institutional memory and safeguarding access to data and information in case of change are not yet organized.

Concerning OH learning infrastructure (0.50), the main focus was on adaptive and generative team learning, the team meetings consisting on information sharing, discussion, and analysis, enabling supportive decision-making and corrective approach. The Initiative aims that the stakeholders involved, namely, the health authorities and institutions, be focused on improving procedures and evolving effectively toward new intervention and approach strategies, but there is some inertia to changing more conservative disciplinary paradigms of health surveillance, even when this change is seen as advantageous and appropriate.

Systematic organization (0.50) assessed team structure, leadership, and focus. The Initiative depends on teamwork. Several teams are to be involved at different organizational and operational levels, although some teams’ structure and objectives need to be further clarified. The Initiative is organized in interrelated and interdependent working packages, which may involve different sectors and disciplines from different fields of expertise within human and animal health. Disciplinarity and transdisciplinarity are being considered in team constitution that is still ongoing. Task-oriented, relationship-oriented, and change-oriented leaderships are present, although unbalanced: informal connections and face-to-face processes are privileged. The OH challenge, the mainstay of the Initiative, is translated into the scientific and developmental questions of the Initiative and the innovation in relation to the state of the knowledge.

Globally, the evaluation conducted indicates that OTC is being designed complying with OH characteristics. The Initiative introduces methodological innovation to which sectorial health authorities may not be completely receptive in practice even though considering the OH approach highly relevant on theoretical grounds. Considerable obstacles are deemed to occur; they need to be both reasoned beforehand and tackled as they arise. For instance, different governmental institutions have valuable data, potentially useful for surveillance, the data being, however, cumbersome to obtain and lacking transparency. Moreover, cysticercosis is a neglected and infrequent disease in Portugal, which may contribute to the general indifference and non-prioritization by the health authorities. However, the already available data suggest the disease burden and the social, ecological, and economic consequences along with the possibility of disease emergence may not be negligible, moreover, considering globalization and increasing human and animal mobility and trade (4, 15). The scientific community, including CYSTINET (European Network on Taeniasis/Cysticercosis), is actively urging to introduce and improve human and porcine cysticercosis surveillance and control practices in Europe (16). New knowledge concerning the national epidemiologic scenario of cysticercosis has already been produced. It was internally and externally disseminated in communications, conferences, and peer-reviewed international publications. The results produced are steps toward the mobilization and harmonization of working methodologies toward an OH model.

## Limitations

The application of the NEOH evaluation framework to the ongoing design of the OTC proved challenging at times. It illustrates an innovative approach to the evaluation framework, this framework being itself pioneer in the context of OH evaluation. The timing of the evaluation allows for introduction of corrections and adjustments in the Initiative to ensure an OH-oriented implementation. However, NEOH tools require a large amount of specific data, some of it not available or feasible at the time, making the evaluation process laborious and hard to accomplish, especially considering the evaluation perspective in this case study. Information was gathered within the designing and coordinating teams that consist of elements from public health and epidemiology, medicine, and academia within human and animal health sectors. Some operational and structural aspects are still being considered and pushed forward. The engagement of actors and stakeholders is not complete. Some teams need structuring and to have their roles and tasks more clearly defined. The work is done primarily on voluntary basis or linked to other academic and non-academic institutional programs.

The scoring may be influenced by the conflict between what the Initiative will achieve if everything works out as planned and what is already achieved, namely, at infrastructure level. Even though the assessment tools seem *a priori* to be more suited for retrospective evaluation, the results of the evaluation provide a valuable contribution to the corrective action needed in the OH Initiative. The communication pathways and sharing mechanisms, key elements of the Initiative, need to be carefully reconsidered and reinforced before advancing further in the implementation. The same applies to team structuring, role definition, and communication between teams, at different organizational and executive levels. This feeds back into corrective action on the planning aspect, further defining and organizing the resources for each objective. Some items of learning and systemic organization should be more adequately assessed once the Initiative is fully implemented. It would be relevant to repeat full evaluation after at least 5 years of project implementation so that changes related to the organization of services, information systems, and institutional relationship can be identified, deeper knowledge has been produced, mechanisms for data information sharing have been implemented, and learning aspects can be effectively recognized.

The NEOH evaluation framework anchors on the system theory and combines descriptive and qualitative assessment with a semiquantitative scoring for the evaluation of the degree and structural balance of OH-ness, the individual analysis of the six assessment tools being also valuable. Putting the Initiative into its context, as a subsystem within the system, allowed a more complete understanding of the Initiative, its relevance, and the impact pathways. The completion of the assessment tools may be subject to bias toward the perception of a particular evaluator; furthermore, considering that this was a self-evaluation process. The review of the scoring criteria by several others evaluators, both internal and external and from different disciplines, helped to address this bias. Summarizing the six assessed OH aspects in a single quantitative and visual element, the OHI and OHR, is attractive as an objective evaluation result in itself and for comparing different Initiatives. Its meaning or interpretation, however, is not yet well defined. A higher OHI does not necessarily indicate a better OH Initiative, so it is difficult to draw conclusions of the results of this case study comparing to others (5). However, this case study is one in a group of case studies, providing the first data on OHI and OHR for various contexts and OH Initiatives. Data of the evaluation framework data from this case study will be compared with other case studies during validation of the framework and in creating benchmarks in the future. Complementary to OH evaluation, conventional evaluation models need to be considered, namely, process evaluation and cost–benefit evaluation, and will provide data to confront with OH-ness evaluation and will provide more detailed analysis on the surveillance planning, implementation and cost–benefit.

Considering the case study presented, NEOH evaluation framework comes forth as an interesting tool also to guide the design of OH Initiatives. In this perspective, operational aspects (OH thinking, OH planning, and OH working) are more tangible to assess. However, infrastructure aspects tools, less applicable, may display relevant information that if disregarded will compromise successful implementation of the Initiative, considering the OH goal.

Evaluation results enable redirecting of the Initiative toward a more OH-oriented perspective. Evaluation results can, moreover, contribute to (i) develop shared guidance and recommendations for surveillance, intervention, prevention, and control of cysticercosis; (ii) be used on Animal and Public Health policy and decision at regional and national level; and (iii) provide insight and an example on how to manage a non-notifiable neglected zoonosis in a European country.

## Conclusion

The taeniasis and cysticercosis health challenge in industrialized countries, namely, Portugal among other European countries, urges OH solutions. This case study explores the reasoning behind the taeniasis- and cysticercosis-integrated surveillance challenge, within the United Nations 2030 agenda, considering the added value of OH for health and welfare in an ecological perspective.

It illustrates the successful application of the NEOH evaluation framework to the ongoing design of OTC in Portugal as a means to ensure adhesion to OH standards. Reflective and corrective action to strengthen team structure, beyond the human health and animal health sector, and communication and information sharing mechanisms is needed, therefore amplifying and clarifying the real scope of the OH concept. Health authorities’ increased awareness is needed for the non-negligible health, social, ecological, and economic issues associated with the disease to further fuel effective sectorial collaboration, networking, and transparency. The institutions need reshaping to better facilitate transdisciplinary processes, promoting a health (human, animal, and environmental)-centered approach. The use of the evaluation framework to guide OH Initiatives design requires that those involved in the process are comfortable with systems thinking to approach complex and dynamic structures. Some items of the assessment tools, namely, the infrastructure tools, may not be applicable at designing stages even though the attempt to complete them enforces the feedback loop leading to reconsideration or amendment of planning issues or other operational aspects. In future, this may evolve toward adjustments or adaptation of the framework to different evaluation contexts, or else, an evaluation framework-based comprehensive OH checklist to guide project development. Further context-specific research will help to clarify and better understand the strengths and weaknesses of the tool in the different settings.

In conclusion, the case study highlights and supports OH assessment also prospectively, that is, guiding and guaranteeing its planning, designing, and implementation in an OH-oriented perspective. Focusing on a systems approach, the use of this tool for prospective and retrospective evaluation and monitoring may prove a valuable addition in future health programs, strategies, and policies.

## Author Contributions

AF and MV conceived and designed the work and drafted the manuscript. AF, MV, and JT carried out the evaluation. AC, DM, and SG contributed to revising the evaluation. All the authors contributed to the analysis and interpretation of data, participated in the revision for important intellectual content, approved the final version to be published, and agreed to be accountable for the content of the work.

## Conflict of Interest Statement

The authors declare that the research was conducted in the absence of any commercial or financial relationships that could be construed as a potential conflict of interest.
